# Work-related musculoskeletal disorders: prevalence, associated factors, and impact on quality of life among kitchen workers in hospitality industry, Bahir Dar City, Northwest Ethiopia, 2023

**DOI:** 10.3389/fpubh.2024.1358867

**Published:** 2024-05-14

**Authors:** Tadiwos Abebaw, Bikes Destaw, Dawit Getachew Yenealem, Amensisa Hailu Tesfaye, Christian Melaku, Yimer Mamaye, Anmut Endalkachew Bezie, Giziew Abere

**Affiliations:** ^1^Department of Occupational Health and Safety, College of Medicine and Health Sciences, Wollo University, Dessie, Ethiopia; ^2^Department of Environmental and Occupational Health and Safety, Institute of Public Health, College of Medicine and Health Sciences, University of Gondar, Gondar, Ethiopia

**Keywords:** work-related musculoskeletal disorders, Nordic, quality of life, SF-36, kitchen workers, Ethiopia

## Abstract

**Introduction:**

Work-related musculoskeletal disorders (WMSDs) are considered major public health problems globally, deteriorating the quality of life of workers in various occupations. Kitchen work is reported as among the occupations most prone to these maladies. Nevertheless, prevalence of WMSDs, contributing factors, and impacts on the quality of life of hospitality industry kitchen workers are insufficiently documented in Ethiopia. Therefore, this study aimed to assess the prevalence of WMSDs, their associated factors, and impact on the quality of life of hospitality industry kitchen workers in Bahir Dar city, Ethiopia.

**Methods:**

An institution-based, cross-sectional study was conducted from 17 April to 17 May 2023. A total of 422 participants were included using a simple random sampling technique. WMSDs were evaluated using an interviewer-administered Nordic standardized questionnaire. The short form-36 questionnaire was used to assess quality of life. The data were collected using the Kobo tool box. SPSS version 26 software was used to perform both bivariable and multivariable binary logistic regression analyses. Independent *t*-tests were used to show the impact of WMSDs on quality of life scales across groups with and without WMSD symptoms.

**Result:**

In this study, the response rate was 98.34% (*n* = 415). The 1-year prevalence of WMSDs among kitchen workers was 82.7% [95% CI: (79.1, 86.3)]. Age group between 30 and 39 years [AOR: 2.81; 95% CI: (1.46–5.41)], job dissatisfaction [AOR: 2.45; 95% CI: (1.34–4.45)], anxiety [AOR: 2.26; 95% CI: (1.12–4.52)], prolonged standing [AOR: 3.81; 95% CI: (1.58–9.17)], and arm overreaching [AOR: 2.43; 95% CI: (1.34–4.41)] were significantly associated factors with work-related musculoskeletal disorders. Work-related musculoskeletal disorders had a significant impact on all quality of life dimensions, in which the mean SF-36 scores of participants with WMSDs were lower than those of their non-WMSD counterparts.

**Conclusion:**

This study revealed that the prevalence of WMSDs was relatively high. Age between 30 and 39 years, job dissatisfaction, anxiety, prolonged standing, and arm overreaching were identified as significant determinants of WMSDs among kitchen workers in hospitality industries. The presence of one or multiple WMSDs, in turn, is associated with worse quality of life dimensions of individuals.

## 1 Introduction

In recent decades, prompt industrialization, technological advancements, and shifts in the global work force structure made work-related musculoskeletal disorders (WMSDs) as a leading costly occupational disease burden contributor (1–3).

Work-related musculoskeletal disorders (WMSDs), also known as cumulative trauma disorders or repetitive strain injuries, are inflammatory or degenerative conditions of the muscles, tendons, ligaments, joints, nerves, bones, and the localized blood circulation system in different body regions presenting with ache, pain, tension, and discomfort that are induced or intensified by work and the immediate environment in which work is performed (4–6). They may occur within weeks, months, or even years prominently elicited following repeated exposure to work place risk factors such as sustained working posture, frequently repeated tasks, heavy weight manual lifts, and others for an extended period of time and rarely due to the emergence of acute injuries after a sudden strain and severe trauma happened (4, 7).

According to the World Health Organization (WHO) report, approximately 1.71 billion people worldwide have musculoskeletal disorders, of which 50–70% comprising workers in various occupations (2, 8). In the United States and United Kingdom, these disorders constitute more than half of all reported occupational illnesses (9, 10). Moreover, it significantly affects low- and middle-income countries (LMICs), including Africa, despite limitations in reporting systems (11). A systematic review stated that in many African countries, the prevalence of any musculoskeletal disease ranged from 15 to 93.6% (12). Studies carried out on the working population of Ethiopia have also shown similar trend with prevalence varying between 47.7 and 81.5% (13–17). As various evidence studies indicated, these figures are likely attributed from the multiple effects of biomechanical, work organizational, psychosocial, and/or other individual factors (6, 7, 18, 19).

Globally, WMSDs cause a paramount impact on employees and organizations, as they lead to sickness absenteeism and decreased productivity, and deleteriously affect the quality of life (QoL) of the working population (4, 20, 21). The WHO defines QoL as an individual’s perceptions of their position in life regarding the context of the culture and value systems in which they live and in relation to their goals, expectations, standards, and concerns, as the relationship between health and illness at work is directly related to QoL (9, 20, 22). Several studies held in different parts of the world suggested that lowered QoL domains were highly associated with having one or more WMSD symptoms (5, 21, 23). A recent global burden of disease report also indicated that WMSDs were among the foremost contributors of disability-adjusted life years (DALYs) in all continents and economies (24, 25). In addition, these illnesses impose enormous costs on society and national health systems across the world, leaving them the second most common and disabling public health problem (3, 5, 7, 10, 26).

Multisite WMSDs are considered a significant health problem among kitchen workers in the hospitality industry (26). Various studies held in different countries which implied its seriousness; for instance, in Taiwan 85.2%, Nepal 60%, and China 47%, prevalence of WMSDs was reported (27–29). A more recent study in Egypt carried out on kitchen workers showed a 90.6% WMSD prevalence in which highly intensified pain on the lower back (64.8%), knee (46.9%), foot (46.1%), neck (29.7%), and shoulders (23.4%) was reported (8). As various studies inferred, the high prevalence of WMSDs among cooks is colossally linked with heavy lifting activities in an awkward posture, long working hours, prolonged standing, highly repetitive tasks, and force overexertion (27–29). Additionally, the quick preparation of quality food for an unpredictable number of customer orders may put excess mental and physical strain that consequently leads to the development of these deficits (26, 29, 30).

In Ethiopia, there has been little progress in addressing the complexity of occupational diseases and employment conditions through the adoption and enactment of occupational health and safety legal provisions (31–33). Nevertheless, ensuring the health and safety of neglected working populations in relative to the general working population, including hospitality industry kitchen workers, is still challenging as these existing judicial tools disregard labor needs and requirements unique to various industries and labor dynamics (34).

Moreover, the sector is not sufficiently explored as it firms as the main auxiliary of tourism activities and contributor of GDP development of the country and provides wide employment opportunities for numerous professions, such as cook, that were not well investigated by the previous local studies on hospitality business employees (35). Evidence has also claimed that psychosocial factors have gained little emphasis as they are evolving over time in parallel with human advancement and has suggested the need for further investigation (18, 23, 28, 29, 36, 37). Despite continuous research on the impact of WMSDs on quality of life (QoL), the issue is still understudied and warrants greater emphasis. Thus, this study aimed to assess the prevalence of WMSDs and their associated factors and impact on QoL among kitchen workers in hospitality industries.

Therefore, the findings may serve as an input for further investigations, particularly on neglected working populations. It may also aid in the development and implementation of tailored WMSD prevention and control programs on contemporarily evolving health hazards such as workplace ergonomics and psychosocial determinants of health to improve QoL and reduce their economic burden in the long run.

## 2 Objectives

### 2.1 General objective

The general objective of this study is to assess the prevalence and associated factors of WMSDs and their impact on QoL among hospitality industry kitchen workers in Bahir Dar City, Amhara Administrative Regional State, Northwest Ethiopia, 2023.

### 2.2 Specific objectives

The specific objectives of this study are to determine the prevalence of WMSDs among hospitality industry kitchen workers in Bahir Dar City, to identify factors associated with WMSDs among hospitality industry kitchen workers in Bahir Dar City, and to assess the impact of WMSDs on the QoL of hospitality industry kitchen workers in Bahir Dar City.

## 3 Methods

### 3.1 Study design and period

An institution-based cross-sectional study was employed from 17 April to 17 May 2023.

### 3.2 Study area

The study was conducted in Bahir Dar City situated at an altitude of 1820 m above sea level. The city is located approximately 578 km north–northwest of Addis Ababa. Bahir Dar City has six subcities, namely, Atse Tewodrose, Dagmawi Menelik, Gish Abay, Fasilo, Tana, and Belay Zeleke. In the city, there are 408 hospitality industries that provide services for customers with 816 kitchen workers, as inferred by the regional and Bahir Dar City culture and tourism bureau and various sector administration offices of each sub city.

### 3.3 Source and study population

All kitchen workers in the hospitality industry found in Bahir Dar City and hospitality industry kitchen workers available during data collection were used as the source and study population, respectively.

### 3.4 Inclusion and exclusion criteria

All kitchen workers in hospitality industries having at least 1 year of experience were included in the study. Meanwhile, hospitality industry kitchen workers who had a previous history of traumatic injuries or accidents other than work, such as car accidents, fall causing musculoskeletal system fractures, and dislocations, MSDs due to other causes not related to work, such as congenital and medical conditions, including surgeries resulting in physical deformity and pregnant women, those who were on sick, annual, and maternity leave were excluded from the study.

### 3.5 Sample size determination

The optimal sample size for the study was determined using a single population proportion formula based on the following assumptions:

*Z* = 95% Confidence interval at α level 5% = 1.96.

Proportion of WMSD prevalence among kitchen workers (*P*) = 50% (0.5). There is no previous study held on hospitality industry kitchen workers in our local context.

Margin of error (*d*) = 0.05 (5%).

Non-response rate = 10%.

The sample size was computed as follows:


$$n=\frac{{\left(Za_{/2}\right)}^{2}P\left(1-P\right)}{d^{2}}$$

$$n=\frac{{\left(1.96\right)}^{2}\times 0.5\left(1-0.5\right)}{{\left(0.05\right)}^{2}}=384.16\approx 384$$

After adding the non-response rate (10%), i.e., 10% percent of 384 were equal to 38.4 ≈ 38, so the total sample size was 384 + 38 = 422.

### 3.6 Sampling technique and procedure

**Figure 1:**
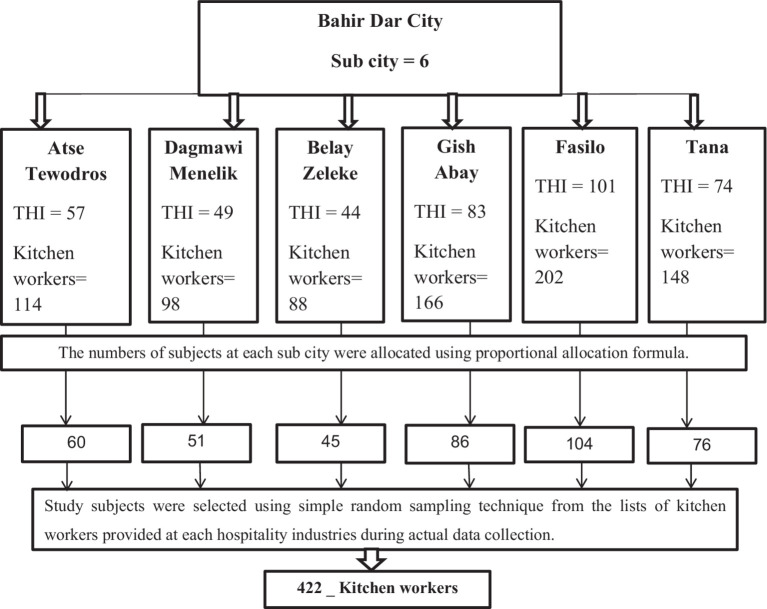
Diagrammatic representation of the sampling procedure.

The needed 422 study subjects were proportionally allocated to each subcity, according to the total number of hospitality industry kitchen workers. A simple random sampling technique was employed to select all the proportionally allocated representative samples based on the list provided by each hospitality business authority during actual data collection, and conclusions were made about the study area (Figure 1).

### 3.7 Operational definition

Hospitality industry: Hospitality industries are service-giving hotels, restaurants, cafeterias, resorts, and lodges (38).

Kitchen workers: Kitchen workers are cookers who were working in hospitality industries.

WMSDs: A musculoskeletal disorder was considered work related if the kitchen workers began to experience the musculoskeletal symptoms after being employed in the kitchen profession and if those symptoms happened only during the working hours in relation to work tasks and not to any recent or past trauma, worsened at work, and relieved during holidays. WMSDs were considered present if the respondent reported the occurrence of work-related musculoskeletal symptoms such as ache, pain, numbness, tingling, or discomfort in any body parts during the last 12 months (5).

Impact of WMSDs on QoL: The effect of WMSDs on the resulting mean score difference on the eight SF-36 QoL domains among participants with WMSDs and without WMSDs (5, 39, 40).

Body mass index (BMI): Weight in kilograms divided by the square of the height in meters (kg/m2) (41). Underweight = BMI < 18.50, Normal range = BMI b/n 18.50–24.99, Overweight = BMI b/n 25.00–29.99, and Obese = BMI ≥30.00.

Job satisfaction: Hospitality industry kitchen workers were considered satisfied with a job when their sum of generic job satisfaction scale score was 32 or above (42).

Job stress: Hospitality industry kitchen workers were considered stressed with a job when their sum of generic job stress scale score was 16 and above, whereas less than 16 was categorized as non-stressed (43).

Physical exercise: Performing any type of physical exercise away from work at least two times/week for 30 min (19).

Repetitive task: Work involving repeating the same motion with little or no variation every few seconds for 2 or more hours (44).

Awkward posture includes working with the neck and/or back bent without support, working with a bent wrist, squatting, and kneeling for 2 or more hours (44).

Prolonged standing: standing for more than 4 h (45).

Alcohol drinking is the consumption of any kind of alcohol by hospitality industry kitchen workers at least two times per week (46).

Cigarette smoking: Smoking at least one stick of cigarette per day (46).

Khat chewing: Chewing khat three times a week (47).

Anxiety: Individuals who scored greater than or equal to 8 for questions related to anxiety in a DAS scale were considered anxious (48).

Sleep disturbance: Individuals who scored less than 5 in the sleep hygiene index were considered to have sleep disturbance (49).

### 3.8 Data collection tools

A structured interviewer-administered questionnaire developed from various literature studies and standardized measurement scales was used. Section I contains sociodemographic variables. Section II contains individual or behavioral characteristics. Section III contains psychosocial variables. Section IV contains workplace environment variables. Section V contains questions regarding WMSDs. Section VI contains questions concerning coping strategies, and Section VII contains questions regarding quality of life (QoL).

The questions used to investigate the sociodemographic, individual, or behavioral and psychosocial and workplace environment were developed from the literature about the potential risk factors for WMSDs and from the standardized Dutch Musculoskeletal Questionnaire (DMQ), Depression and Anxiety using Depression Anxiety Stress Scale (DASS), Job stress scale, Job satisfaction scale, and Step Sleep Hygiene Index.

A standardized Nordic questionnaire was used regarding the WMSD prevalence in the different body parts. To assess the impact of WMSDs on an individual’s quality of life, the 36-item Short Form Health Survey (SF-36) questionnaire was used, and it has eight scales in which scores are described as weighted sums of the questions in each section that range from 0 to 100. Lower scores indicate a lower quality of life dimension, and higher scores indicate a good quality of life dimension. Sections include: Physical functioning consists of 10 items concerning the subject level of performing vigorous activities, such as running, lifting heavy objects, and participating in strenuous sports; moderate activities, such as moving a table, pushing a vacuum cleaner, bowling or playing, lifting or carrying groceries, climbing several flights of stairs, climbing one flight of stairs, bending, kneeling or stooping, walking more than a mile, walking several blocks, walking one block, and bathing or dressing;

Body pain consists of two items related to subjects’ experience of body pain and how much did pain interfere with their normal work, including both work outside the home and housework.

General health perception consists of five items concerning subjects’ current experience of sickness and health status in relation to others and how they expect their health status in their future life.

Physical role functioning consists of four items related to subjects’ amount of time spent on work or other activities, level of accomplishment, and difficulties in performing their work or other activities.

Emotional role functioning consists of three items concerning the amount of time subjects spent on work or other activities and level of accomplishment and how they carefully perform work activities than usual.

Social role functioning consists of two items regarding subjects’ extent of physical health or emotional problems interfered with their normal social activities with family, friends, neighbors, or groups and how much of the time their physical health or emotional problems interfered with their social activities, such as visiting friends or relatives.

Mental health consists of five items regarding subjects’ life experiences and their perception and emotional reactions to their life events.

Energy/vitality/fatigue consists of four items related to the experiences of subjects and incorporates questions focusing on feelings of being worn out, tired, and pep experiences.

The remaining item is the health transition item (HTI), which focuses on individuals’ perceptions of their current health status relative to the previous year.

### 3.9 Data collection procedure and quality control

Interviewer-administered data were collected using kobo tool box software by five personnel with B.Sc. in health professions who were guided by two MPH supervisors.

Before the data collection, data collectors and supervisors were given 2 days of training on the study’s purpose, data collection techniques, data collection tools, respondent approach, data confidentiality, and respondent rights. The assessment tools were translated into local language/Amharic for easy communication of data collectors and participants’ understanding of enquire.

A pretest was conducted on 5% (21 questionnaires) of hospitality industry kitchen workers in Gondar city, and appropriate modifications were made. Brief explanation about the studies’ purpose was given to the respondents before preceding an interview, and finally, the data collectors conducted interviews with those who gave their verbal consent to participate in the study.

Accordingly, the reliability test results revealed that Cronbach’s alpha coefficient for each QoL scale was as follows: physical functioning (Cronbach’s *∝* = 0.968), role limitations due to physical health problems (Cronbach’s ∝ = 0.863), role limitations due to emotional health problems (Cronbach’s *∝* = 0.947), pain (Cronbach’s *∝* = 0.931), social functioning (Cronbach’s *∝* = 0.928), emotional/mental health (Cronbach’s *∝* = 0.852), vitality (Cronbach’s *∝* = 0.817), and general health (Cronbach’s *∝* = 0.861).

Actual data collection was held based on the findings of the pretest. The supervisors were monitoring and examining the completeness and accuracy of the collected data every day. The gathered data were cleaned up and cross-checked before analysis.

### 3.10 Data processing and analysis

Quantitative data were checked, edited, and exported to SPSS version 26 for analysis. Descriptive statistics of the sociodemographic, individual, and psychosocial characteristics, work organizational and physical or ergonomic characteristics, prevalence of overall WMSDs and at each specific body site, and quality of life dimension scores were computed.

The descriptive findings were summarized using the mean, standard deviation, median, and interquartile range for quantitative data and frequencies and percentages for categorical variables and are presented using tables and graphs.

A comparison between the two groups of kitchen workers who reported WMSDs and not reported WMSDs over the last 12 months to detect the basic statistical mean difference between the independent QoL dimensions was evaluated using an independent t-test. A two-sided *p*-value ≤0.05 significance level was used for this study. Additionally, the multicollinearity assumption was checked through the variance inflation factor (VIF), and all variables showed a VIF < 1.31.

A binary logistic regression analysis model was used to show the presence of a statistically significant association between WMSDs and independent variables (sociodemographic, individual, psychosocial, work organizational, and ergonomic factors) with a 95% CI, and all variables in the bivariable with a p-value less than 0.2 were included in the multivariable logistic regression analysis, and a p-value of less than 0.05 was considered statistically significant. Goodness of fit was checked by the Hosmer and Lemeshow test (*p*-value = 0.986), which showed that the model fit well.

## 4 Results

### 4.1 Sociodemographic characteristics of participants

A total of 422 participants were recruited with a 98.34% (415) response rate. The majority, 396 (95.4%), of the participants were female. The mean (±SD) age of the participants was 26 ± 5 years. The median monthly salary of participants was 3,500 ETB with an interquartile range (IQR) of 3,000 to 4,500 birr. Over half of the participants also had work experience of less than or equal to 4 years (Table 1).

**Table 1:** Sociodemographic characteristics of hospitality industry kitchen workers in Bahir Dar city, Northwest Ethiopia, 2023 (*n* = 415).

Variables	Categories	Frequency	Percent (%)
Sex	Male	19	4.6
	Female	396	95.4
Age	18–29	214	51.6
	30–39	166	40.0
	> = 40	35	8.4
Marital status	Single	245	59.8
	Married	124	29.9
	Divorced	33	7.7
	Widowed	14	3.4
Education	Unable to read and write	69	16.6
	Read and write	29	7.0
	1–8	145	34.9
	9–12	100	24.1
	12 + (certificate/diploma/degree)	72	17.3
Monthly salary	<=3,500	253	61.0
	>3,500	162	39.0
Specific work experience	<=4 years	239	57.6
	>4 years	176	42.4

### 4.2 Individual or behavioral and psychosocial factors

Out of 415 study participants, 288 (69.4%) had a BMI of 18.50–24.99 kg/m^2^. Only 54 (13.0%) had a habit of engaging in physical exercise at least 2 days per week. In higher proportions, 382 (92%) of participants were non-alcohol drinkers.

Regarding psychosocial factors, nearly all, 401 (96.6%), of the participants reported job stress, as opposed to sleep disturbance in which equal number of participants had not experienced any. On the other hand, the majority of respondents, 301 (72.5%), were not satisfied with their job. Over half, 275 (66.3%), of the participants had no anxiety symptoms (Table 2).

**Table 2:** Behavioral and psychosocial factors of hospitality industry kitchen workers in Bahir Dar city, Northwest Ethiopia, 2023 (*n* = 415).

Variables	Categories	Frequency	Percent (%)
Cigarette smoking	Yes	0	0.00
	No	415	100
Khat chewing	Yes	0	0.00
	No	415	100
Alcohol drinking	Yes	33	8.0
	No	382	92.0
Physical exercise	Yes	54	13.0
	No	361	87.0
Body mass index (BMI)	Underweight	18	4.3
	Normal	288	69.4
	Overweight	109	26.3
Systemic illness	Yes	33	8.0
	No	382	92.0
Job stress	Stressed	401	96.6
	Not stressed	14	3.4
Job satisfaction	Satisfied	114	27.5
	Unsatisfied	301	72.5
Depression	Depressed	31	7.5
	Not depressed	384	92.5
Anxiety	Anxious	140	33.7
	Not anxious	275	66.3
Sleep disturbance	Yes	14	3.4
	No	401	96.6

### 4.3 Work-environment factors

In terms of work-environment factors, over three-fourth, 316 (76.9%), of the participants spent more than 8 h per day in work. None of the participants had taken training concerning on issues of ergonomics. A large proportion, 375 (90.4%), of the respondents reported prolonged standing in their kitchen work; similarly, almost all 387 (93.2%) of the participants also reported that their job involved repetitive work. Participants reported that their cooking task needed overreaching movements of arms also accounted for 206 (48.8%). However, the majority, 378 (91.1%), of the respondents’ kitchen task allowed them to handle loads less than or equal to 5 kg of weight (Table 3).

**Table 3:** Work environment factors of hospitality industries in Bahir Dar city, Northwest Ethiopia, 2023 (*n* = 415).

Variables	Categories	Frequency	Percent (%)
Working hour	<=8	99	23.1
	>8	316	76.9
Shift work	Yes	105	25.3
	No	310	74.7
Ergonomics training	Yes	0	0.00
	No	415	100
Prolonged standing	Yes	375	90.4
	No	40	9.6
Repetitive work	Yes	387	93.2
	No	28	6.74
Awkward posture	Yes	309	74.5
	No	106	25.5
Loads lifting/carrying/pushing (kg)	<=5	378	91.1
	>5	37	8.9
Arm overreaching	Yes	206	48.8
	No	216	51.2
Force overexertion	Yes	38	9.2
	No	377	90.8

### 4.4 Prevalence of work-related musculoskeletal disorders among hospitality industry kitchen workers

The findings of this study revealed that among the 415 kitchen workers, 343 participants reported WMSD complaints in any body parts. The overall prevalence of self-reported WMSDs over the last 12 months was 82.7% [95% CI: (79.1, 86.3)], and the past 1-week prevalence was 71.3% (n = 308) [95% CI: (66.7, 75.8)].

**Figure 2:**
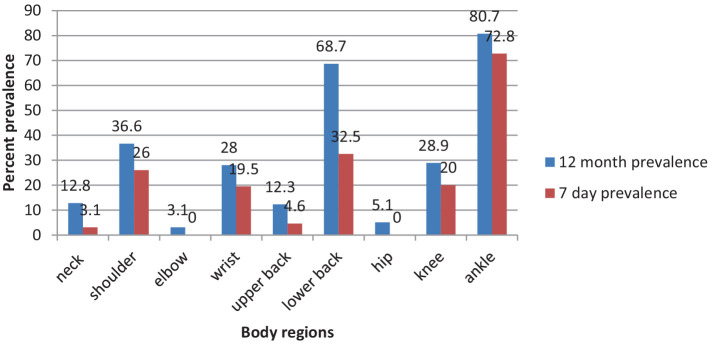
Prevalence of WMSDs by body regions in the past 12 months and 7 days among the studied kitchen workers (*n* = 415).

The present study revealed that ankle or foot pain (80.7%) [95% CI: (76.6–84.8)] was the most prevalent WMSD among kitchen workers over the past 12 months, and lower back pain (68.7%) [95% CI: (64.3–73.3)] was the second most common WMSD complaint, followed by shoulder pain (36.6%) [95% CI: (31.6–41.0)], knee pain (28.9%) [95% CI: (24.4–32.5)], and wrist pain (28%) [95% CI: (23.2–32.3)]. In low proportions, neck (12.8%), upper back (12.3%), hip/thigh (5.1%), and elbow (3.1%) constitute the least WMSD-affected category of body regions (Figure 2).

**Figure 3:**
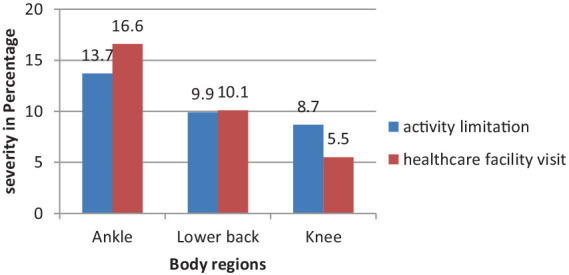
Body regions severely affected by WMSDs in the past 12 months among the studied kitchen workers (*n* = 415).

In the previous 12 months, some of those WMSD-affected sites prevented participants from doing their normal work at home or away from home, mainly due to ankle or foot pain (13.7%), lower back pain (9.9%), and knee pain (8.7%). Moreover, kitchen workers’ hospital visits related to WMSD troubles also predominantly resulted from ankle or foot (16.6%), lower back (10.1%), and knee (5.5%) musculoskeletal problems (Figure 3).

Approximately 81 (19.5%) of the participants had no more than two WMSD-affected sites. In equal number, 106 (25.5%) of the respondents had trouble with three and four WMSDs. Fifty (12%) of the participants had five or more WMSD symptoms at any body parts (Figure 3).

### 4.5 Self-adopted WMSD coping up mechanisms

The results of this study indicated that a major proportion (88.1%) of kitchen workers with WMSD symptoms at any body parts preferred to take sufficient rest at work or away from work to obtain adequate relief and return to their work with full capacities. Similarly, those who had performed position modifications and relied on homemade remedies accounted for approximately 93.58 and 92.7%, respectively (Table 4).

**Table 4:** Self-adopted management strategies for work-related musculoskeletal complaints of hospitality industry kitchen workers in Bahir Dar city, Northwest Ethiopia, 2023 (*n* = 415).

Variables	Categories	Frequency	Percent (%)
Didn’t do any things	Yes	72	82.7
	No	343	17.1
Taking sufficient rest	Yes	302	88.1
	No	41	11.9
Reduced working hours	Yes	14	4.08
	No	329	95.9
Visited a physician	Yes	85	24.8
	No	258	75.2
Stop attending to customers if it causes or worsens discomfort	Yes	43	12.5
	No	300	87.5
Modifying the position to be comfortable	Yes	321	93.58
	No	22	6.41
Take homemade management	Yes	318	92.7
	No	25	7.28

### 4.6 Factors associated with work-related musculoskeletal disorders

In the bivariable logistic regression analysis, age, BMI, physical exercise, job dissatisfaction, anxiety, repetitive work, prolonged standing, and arm overreaching were factors associated with WMSDs. However, after controlling the confounding variables in the multivariable binary logistic regression analysis, age group between 30 and 39 years, job dissatisfaction, anxiety, prolonged standing, and arm overreaching were found to be significant variables associated with WMSD problems at various body segments (Table 5).

**Table 5:** Bivariable and multivariable binary logistic regression analysis of factors associated with work-related musculoskeletal disorders among hospitality industry kitchen workers in Bahir Dar city, Northwest Ethiopia, 2023 (*n* = 415).

Variables	Categories	WMSDs		COR (95% CI)	AOR (95% CI)
		Yes	No		
Age	18–29	164	50	1	1
	30–39	151	15	**3.06 (1.65–5.69)**	2.81 (1.46–5.41)**
	> = 40	28	7	1.22 (0.50–2.96)	1.27 (0.48–3.35)
BMI	Underweight	11	7	1	1
	Normal	238	50	**3.02 (1.11–8.19)**	2.04 (0.67–6.24)
	Overweight	94	15	**3.98 (1.33–11.89)**	1.93 (0.56–6.55)
Physical exercise	Yes	38	16	1	1
	No	305	56	**2.29 (1.19–4.39)**	1.44 (0.69–2.98)
Job satisfaction	Satisfied	84	30	1	1
	Unsatisfied	259	42	**2.20 (1.29–3.73)**	2.45 (1.34–4.45)**
Anxiety	Anxious	127	13	**2.66 (1.40–5.05)**	2.26 (1.12–4.52)*
	Not anxious	216	59	1	1
Repetitive work	Yes	326	61	**3.45 (1.10–11.62)**	0.99 (0.21–4.88)
	No	17	11	1	1
Prolonged standing	Yes	319	56	**3.79 (1.89–7.59)**	3.81 (1.58–9.17)**
	No	24	16	1	1
Arm overreaching	Yes	182	21	**2.74 (1.58–4.76)**	2.43 (1.34–4.41)**
	No	161	51	1	1

WMSDs, work-related musculoskeletal disorders, COR, crude odds ratio, CI, confidence interval; AOR, adjusted odds ratio; ^*^P-value < 0.05; ^**^P-value < 0.01; Hosmer and Lemeshow test = 0.986.

Accordingly, the probability of developing WMSDs was 2.81 times higher among participants aged between 30 and 39 years than among their younger counterparts [AOR: 2.81; 95% CI: (1.46–5.41)]. Participants who had dissatisfaction with their job were 2.5 times at high risk of developing WMSDs compared with those who had satisfaction with their job [AOR: 2.45; 95% CI: (1.34–4.45)]. The odds of developing WMSDs were 2.25 times higher among participants who had experienced anxiety symptoms than among those who had not experienced anxiety symptoms [AOR: 2.26; 95% CI: (1.12–4.52)]. The likelihood of developing WMSDs was 3.98 times higher among participants who had been exposed to prolonged standing compared with those who had not been exposed [AOR: 3.81; 95% CI: (1.58–9.17)]. Participants whose work persuaded overreaching of arms were 2.4 times more likely to develop WMSDs than those whose work did not persuade overreaching of arms [AOR: 2.43; 95% CI: (1.34–4.41)] (Table 5).

### 4.7 Impact of WMSDs on quality of life (QoL)

Concerning the mean values of QoL scales in the SF-36 health survey questionnaire, the mean ± SD score of physical functioning was 66.98 ± 11.83, that of role limitations due to physical health problems was 77.59 ± 13.43, that of role limitations due to emotional problems was 73.21 ± 9.69, that of vitality was 51.02 ± 5.36, that of mental health was 70.62 ± 5.93, that of social functioning was 78.73 ± 15.43, that of bodily pain was 68.73 ± 18.04 and that of general health was 68.61 ± 8.32.

The impact of WMSDs on each SF-36 quality of life domain was observed using an independent t-test, and the mean values were lower with respondents with WMSDs, and the computed *p*-values were less than 0.05 for all QoL scales (Table 6).

**Table 6:** Impact of work-related musculoskeletal disorders on the quality of life of hospitality industry kitchen workers in Bahir Dar city, Northwest Ethiopia, 2023 (*n* = 415).

Scales	All kitchen workers (*n* = 415) Mean ± SD	With WMSDs (*n* = 343) Mean ± SD	Without WMSDs (*n* = 72) Mean ± SD	*P*-value
Physical functioning	66.98 ± 11.83	62.94 ± 8.05	86.25 ± 7.05	0.000
Role limitations due to physical health	77.59 ± 13.43	73.57 ± 10.65	96.7 ± 7.56	0.000
Role limitations due to emotional problems	73.21 ± 9.69	72.57 ± 9.56	76.27 ± 9.75	0.003
Energy/Fatigue /Vitality	51.02 ± 5.36	49.73 ± 5.97	51.29 ± 5.19	0.025
Emotional well-being (Mental health)	70.62 ± 5.93	69.95 ± 5.83	73.81 ± 5.4	0.000
Social Functioning	78.73 ± 15.43	75.67 ± 14.33	92.88 ± 12.25	0.000
Pain	68.73 ± 18.04	64.55 ± 15.56	88.64 ± 15.69	0.000
General health	68.61 ± 8.32	66.31 ± 6.58	79.57 ± 6.89	0.000

The final score of the SF-36 ranges from 0 to 100, with higher scores indicating higher levels of function and/or better health. ^*^p < 0.05, ^**^p < 0.01, ^***^p < 0.001. *p*-values were calculated using an independent *t* test.

## 5 Discussion

The prevalence of WMSDs in this study was 82.7% [95% CI: (79.1, 86.3)]. The middle age (30–39 years) group, job dissatisfaction, anxiety, prolonged standing, and arm overreaching were identified as risk factors for work-related musculoskeletal disorders among kitchen workers in Bahir Dar’s hospitality industry.

Furthermore, a significant difference in the mean scores of the eight quality of life dimensions between participants with and without WMSD symptoms was observed.

The magnitude of WMSDs in the present study was comparable to the study findings conducted among cooks in Taiwan (85.2%) (28). This consistent prevalence could be due to similarity of activities dominantly prevailing in kitchen work, and WMSDs affect various nations’ food-serving establishment workers irrespective of the difference in living standards and development status (8, 50, 51).

In contrast, the finding of this study was higher than the study findings carried out on kitchen workers in southern India (67.5%) (36) and Nepal (45.3%) (29). The possible reason for this disparity might be that kitchen workers in the present study were exposed to a more intensive manual working process coupled with longer standing working hours. Additionally, women were the dominant workforce, and in addition to time spent in household activities, biologically, they are more susceptible to MSDs due to high hormonal fluctuations, reduced aerobic power, and muscle strength (52).

The other reason for this relatively high prevalence of WMSDs might be due to the prevailing poor occupational health and safety services in Ethiopia, including inadequate health and safety professionals, personal protective equipment (PPE), modern ergonomic aids, and training (17, 53). Furthermore, in the Indian study (36), it was observed that the younger age groups and participants with considerable job satisfaction were taking the highest proportions that might contribute to the lower magnitude of WMSDs in contrast to the current study, where relatively older age groups and participants who were dissatisfied with their job constituted larger proportions.

On the other hand, the current study finding was lower than the study findings held among pastry chefs and kitchen workers in Malaysia (92.3%) (26) and Egypt (90.6%) (8). The possible reason for these variations might be the difference in proportions of age groups and experience of participants included. For instance, in the Malaysian study, participants had a minimum of 10 years of work experience; similarly, in the Egyptian study, over three-fourth of participants had more than 10 years of work experience, and the majority of respondents were also above 30 years of age. In the present study, younger age and participants with fewer years of work experience were included.

Despite the difference in the prevalence of WMSDs, similar studies support the present bodily site-specific findings in this similar and other related study population.

The magnitude of ankles/feet pain (80.7%) in the previous 12 months was in line with the finding in Malaysia (76.9%) conducted among pastry chefs as confined in kitchen work in both studies involved long standing and working hours that imparted plantar pressure and reduced the pain threshold on the feet (54).

Conversely, the magnitude of ankle/feet pain in this study was higher than studies conducted in Turkey (29%) (55), Malaysia (59.5%) (56), Nepal (27.5%) (29), Taiwan (42.8%) (57), Egypt (46.1%) (8), and Ethiopia (41.3%) (17). With regard to the Taiwanese study, this discrepancy could be justified as over one-third of the respondents (44.4%) preferred to self-manage the pain in the ankles and 17.8% were seeking alternative medicine, such as massage and physical therapies. While in the current study, only 16.6% of the respondents with ankle troubles preferred to visit healthcare facilities.

The result of the present study in terms of low back musculoskeletal problem (68.7%) was in line with a study carried out in India (65.8%) (36) in which it exclusively focused on male kitchen workers and nearly all of the participants had less than a daily working hour of eight as well as the majority also had work experience of less than or equal to 5 years, so these factors might make the result comparable with the finding of the present study.

In contrary, the magnitude of low back trouble was higher compared with studies held in Malaysia [(55.8), (52.4%)] (26, 56), Turkey (26%) (55), Taiwan [(40.1%), (56.9%), (52%)] (18, 28, 57), Egypt (53.1) (8), and Ethiopia (53.8) (17). This disparity might be attributed to participants’ preference for site-specific WMSD management strategies, and either medical or self-adopted coping mechanisms were also enhanced in parallel with advancements in educational levels, as illustrated in the Turkish study.

With regard to the prevalence of wrist pain, the result of the current study (28%) was disproportionately higher compared with the exact similar findings in both Nepal (29) and Bangladesh, where only 5% of the participants were reported (58). This discrepancy might be attributed to the fact that the proportion of men was over three-fourths in the Banglans study, whereas women were the dominant workforce in this study, and proness to wrist pain might be favored from the repetitive nature of kitchen work, as it causes nerve entrapments at this site beyond the difference in naturally predisposing factors in comparison to men (52, 59).

Nevertheless, the magnitude of this wrist pain was lower than that in studies conducted in Malaysia [(57.7%), (46.8%)] (26, 56), Taiwan [(46.5%), (38.2%)] (18, 57), and Ethiopia (51.6%) (17). The possible explanation for this disparity might be the difference in ways of work organization, for instance, in the Malaysian (26) study, kitchen tasks were chiefly characterized by lifting, carrying, or pushing heavy loads that are greater than 5 kg of weight and force overexertion in manipulating tools, and except for the numeric explanation, the possible justification is also the same for the Taiwanese study (18).

In summary, the difference in the overall or site-specific magnitude of WMSDs between different study findings might be attributed from the difference in the distribution of sociodemographic, psychosocial, and work environment factors.

In this study, it was found that age cohort of 30–39 years, job dissatisfaction, anxiety, prolonged standing, and arm overreaching were identified as risk factors of work-related musculoskeletal disorders. Participants aged between 30 and 39 years were more likely to develop WMSDs than lower age groups.

This finding is consistent with studies performed in India (36), Bangladesh (58), Egypt (8), and Ethiopia (17, 53). This could possibly be explained by the fact that the biological structures, particularly the musculoskeletal system, may degenerate as age increases in addition to loss of tissue strength and diminution of bone, which may induce pain, and individuals who are in the mid-thirties also prominently experience WMSD symptoms in the form of back pain (52, 60).

The other risk factor having a significant association with WMSDs was job dissatisfaction. This finding is supported by studies conducted in Ethiopia (17, 53), China (61), and New Zealand (62). The possible reason might be that workers who feel unsatisfied with their work are prone to develop stress, which leads to frequent arterial spasms and muscle tension, which may initiate pain in the musculoskeletal system (62–64).

Being anxious was also significantly associated with WMSDs, and a study in Chile (23), Iran (65), Italy (66), and China (61) supported this finding. A possible explanation might be that anxious individuals overreact to potentially dangerous situations that lead to nonadaptive responses, and individuals’ perception of pain may be intensified, which impairs circulation and the supply of oxygen to tissues and halts repair processes; as a result, it increases the risk of WMSDs (67).

Moreover, this study revealed that prolonged standing had a significant association with WMSDs. This finding was in accordance with findings in Ethiopia (68), Malaysia (26), Iran (65), and Bangladesh (58). The corresponding explanation might be undue pressure on tissues, lumbar spine stress, and muscle stiffness created due to long standing hours that eventually leads to musculoskeletal system fatigue and the development of WMSDs (68).

Furthermore, arm overreaching movements were identified as significantly associated risk factors for WMSDs. For instance, studies conducted in Malaysia (26) and Ethiopia (17, 53) noted this bodily movement as one of the most contributing WMSDs harboring risk factors. This might be explained by the fact that arm overreaching movements are inalienable to the tendons, ligaments, joints, and muscles in a neutral posture and result in unnecessary pressure (19, 53), consequently leading to the development of WMSDs.

With regard to the impact of WMSDs on the quality of life of kitchen workers, respondents who developed WMSD complaints had significantly diminished quality of life scores in all SF-36 functioning scales compared with those without WMSD complaints.

This finding agrees with studies in India (40), Brazil (69), Chile (23), and Egypt (5) performed among light engineering maintenance personnel, patients, and teachers, where all eight components had a significant relation with the presence of WMSD-affected sites. In addition, the findings of the present study were also supported by similar studies conducted in Brazil (70), Turkey (71), and Saudi Arabia (39) among dockworkers, teachers, and cashiers, respectively, in which the mean scores of 5 and more of the 8 functioning scales for individuals with WMSDs were detrimentally lower compared with their non-WMSD counterparts. This implies that kitchen workers with WMSDs would have worse quality of life.

## 6 Limitation and strength

Recall bias may have occurred, as the study is based on the previous 12-month symptom experience. Despite, this study is the first to assess the quality of life of kitchen workers in the hospitality sector.

## 7 Conclusion

This study revealed that the prevalence of WMSDs was relatively high among kitchen workers in the Bahir Dar hospitality industry. The various risk factors such as age (30–39 years), job dissatisfaction, anxiety, prolonged standing, and arm overreaching were identified as significant risk factors. Workers with WMSDs had a demonstrably lower QoL across all eight measured dimensions. Tailored strategies to reduce the magnitude of WMSDs and their impact on QoL crucially should focus on ergonomics training, avoidance of prolonged standing and arm overreaching movements, and maintenance of good psychosocial health.

## Data availability statement

The raw data supporting the conclusions of this article will be made available by the authors, without undue reservation.

## Ethics statement

The studies involving human participants were reviewed and approved by the University of Gondar Ethical approval Committee with Re. No: IPH 2501/2023. Permission was obtained from various official public administrators and hospitality industry managers. All procedures were conducted based on the Helsinki declaration. The participants were informed about the purpose of the study, the importance of their participation and right to withdraw at any time and provided their verbal consent to participate in this study.

## Author contributions

TA: Conceptualization, Formal analysis, Funding acquisition, Investigation, Methodology, Software, Supervision, Validation, Visualization, Writing – original draft, Writing – review & editing. BD: Conceptualization, Methodology, Supervision, Validation, Visualization, Writing – review & editing. DY: Conceptualization, Methodology, Supervision, Visualization, Writing – review & editing. AT: Conceptualization, Methodology, Supervision, Visualization, Writing – review & editing. CM: Methodology, Supervision, Visualization, Writing – review & editing, YM: Methodology, Supervision, Visualization, Writing – review & editing. AB: Methodology, Supervision, Visualization, Writing – review & editing. GA: Conceptualization, Methodology, Supervision, Validation, Visualization, Writing – review & editing.
